# Supplementary material for: Mechanobiology and Primary Cilium in the Pathophysiology of Bone Marrow Myeloproliferative Diseases
Source: Int J Mol Sci. 2024 Aug 14;25(16):8860. doi: 10.3390/ijms25168860 (PMC11354938; doi:10.3390/ijms25168860)
Supplement: Supplementary file 1 [file ijms-25-08860-s001.zip › ijms-3093454-supplementary.pdf]

Table S1: Diagnostic criteria for essential thrombocythemia (ET) and post-essential thrombocythemia myelofibrosis (post-ET MF) according to ICC guidelines [6].

The diagnosis of ET requires all major criteria or the first 3 major criteria and the minor criteria. The diagnosis of post-ET MF is defined by the two required criteria and at least two additional criteria. [a]Three or more megakaryocytes lying adjacent without other BM cells in between; in most of these rare clusters < 6 megakaryocytes may be observed, increase in huge clusters (> 6 cells) accompanied by granulocytic proliferation is a morphological hallmark of pre-PMF. [b]Very rarely a minor increase in reticulin fibers may occur at initial diagnosis (MF-1). [c] It is recommended to use highly sensitive assays for *JAK2*V617F (sensitivity level < 1%) and *CALR* and *MPL* (sensitivity level 1–3%)—in negative cases, consider a search for non-canonical *JAK2* and *MPL* mutations. [d]Assessed by cytogenetics or sensitive NGS techniques. [e]Reactive causes of thrombocytosis include a variety of underlying conditions like iron deficiency, chronic infection, chronic inflammatory disease, medication, neoplasia, or history of splenectomy.

	ET		Post-ET MF
**Major criteria**	-Platelet count ≥ 450 × 10^9/L<br>-Bone marrow biopsy showing proliferation mainly of the megakaryocytic lineage, with increased numbers of enlarged, mature mega-karyocytes with hyperlobulated staghorn- like nuclei, infrequently dense clusters[a]; no significant increase or left shift in neutrophil granulopoiesis or erythropoiesis; no relevant BM fibrosis[b]<br>-Diagnostic criteria for *BCR::ABL1* positive chronic myeloid leukemia, polycythemia vera, primary myelofibrosis, or other myeloid neo- plasms are not met<br>-*JAK2*, *CALR*, or *MPL* mutation[c]	**Required criteria**	-Previous established diagnosis of ET<br>-Bone marrow fibrosis of grade 2 or 3 (MF-2 orMF-3)
**Minor criteria**	-Presence of a clonal marker[d] or absence of evidence of reactive thrombocytosis[e]	**Additional criteria**	-Anemia (i.e., below the reference range given age, sex, and altitude considerations) and a > 2 g/dL decrease from baseline hemoglobin concentration<br>-Leukoerythroblastosis<br>-Increase in palpable splenomegaly of > 5 cm from baseline or the development of a newly palpable splenomegaly<br>-Elevated lactate dehydrogenase level above thereference range<br>-Development of any 2 (or all 3) of the following constitutional symptoms: >10% weight loss in 6 months, night sweats, unexplained fever (> 37.5 °C)

Table S2: Diagnostic criteria for early/pre-fibrotic and overt primary myelofibrosis (PMF) according to ICC guidelines [6]. The diagnosis of pre-PMF or overt-PMF requires all 3 major criteria and at least 1 minor criterion confirmed in 2 consecutive determinations. [a]Morphology of megakaryocytes in pre-PMF and overt PMF usually demonstrates a higher degree of megakaryocytic atypia than in any other MPN-subtype; distinctive features of megakaryocytes include small to giant megakaryocytes with a prevalence of severe maturation defects (cloud-like, hypolobulated and hyperchromatic nuclei) and presence of abnormal large dense clusters(mostly > 6 megakaryocytes lying strictly adjacent). [b]It is recommended to use highly sensitive assays for *JAK2* V617F (sensitivity level < 1%) and *CALR* and *MPL* (sensitivity level 1–3%)—in negative cases, consider searching for non-canonical *JAK2* and *MPL* mutations. [c]Assessed by cytogenetics or sensitive NGS techniques; detection of mutations associated with myeloid neoplasms (e.g., *ASXL1*, *EZH2*, *IDH1*, *IDH2*, *SF3B1*, *SRSF2*, and *TET2* mutations) supports the clonal nature of the disease. [d]Minimal reticulin fibrosis (grade 1) secondary to infection, autoimmune disorder or other chronic inflammatory conditions, hairy cell leukemia or another lymphoid neoplasm, metastatic malignancy, or toxic (chronic) myelopathies. [e]Monocytosis can be present at diagnosis or develop during the course of PMF; in these cases, a history of MPN excludes CMML, whereas a higher variant allelic frequency for MPN-associated driver mutations is supporting the diagnosis of PMF with monocytosis rather than CMML.

	Early/pre-fibrotic—PMF	Overt—PMF
**Major criteria**	-Bone marrow biopsy showing megakaryocytic proliferation and atypia[a], bone mar- row fibrosis grade < 2, increased age-adjusted BM cellularity, granulocytic prolifera-tion, and (often) decreased erythropoiesis<br>-*JAK2*, *CALR*, or *MPL* mutation[b] or presence of another clonal marker[c] or absence ofreactive bone marrow reticulin fibrosis[d]<br>-Diagnostic criteria for *BCR::ABL1* positive chronic myeloid leukemia, polycythemiavera, essential thrombocythemia, myelodysplastic syndromes, or other myeloid neoplasms[e] are not met	-Bone marrow biopsy showing megakaryocytic proliferation and atypia[a], accompaniedby reticulin and/or collagen fibrosis grades 2 or 3<br>-*JAK2*, *CALR*, or *MPL* mutation[b] or presence of another clonal marker[c] or absence ofreactive bone marrow reticulin fibrosis[d]<br>-Diagnostic criteria for *BCR::ABL1* positive chronic myeloid leukemia, polycythemiavera, essential thrombocythemia, myelodysplastic syndromes, or other myeloid neoplasms[e] are not met
**Minor criteria**	-Anemia not attributed to a comorbid condition<br>-Leukocytosis $\geq 11 \times 10^9$/L<br>-Palpable splenomegaly<br>-Lactate dehydrogenase level above the reference range	-Bone marrow biopsy showing megakaryocytic proliferation and atypia[a], accompaniedby reticulin and/or collagen fibrosis grades 2 or 3<br>-*JAK2*, *CALR*, or *MPL* mutation[b] or presence of another clonal marker[c] or absence ofreactive bone marrow reticulin fibrosis[d]<br>-Diagnostic criteria for *BCR::ABL1* positive chronic myeloid leukemia, polycythemiavera, essential thrombocythemia, myelodysplastic syndromes, or other myeloid neoplasms[e] are not met<br>-Anemia not attributed to a comorbid condition<br>-Leukocytosis $\geq 11 \times 10^9$/L<br>-Palpable splenomegaly<br>-Lactate dehydrogenase level above the reference range<br>-Leukoerythroblastosis

Table S3: Diagnostic criteria for polycythemia vera (PV) and post polycythemia vera myelofibrosis (post-PV MF) according to ICC guidelines [6].

	PV		Post-PV MF
**Major criteria**	-Elevated hemoglobin concentration or elevated hematocrit or increased red blood cell mass<br>-Bone marrow biopsy showing age-adjusted hypercellularity with trilineage proliferation (panmyelosis), including prominent erythroid, granulocytic, and increase in pleomorphic, mature megakaryocytes without atypia<br>-Presence of JAK2 V617F or JAK2 exon 12 mutation	**Required criteria**	-Previous established diagnosis of PV
**Minor criteria**	-Subnormal serum erythropoietin level	**Additional criteria**	-Anemia (i.e., below the reference range given age, sex, and altitude considerations) or sustained loss of requirement of either phlebotomy (in the absence of cytoreductive therapy) or cytoreductive treatment for erythrocytosis<br>-Leukoerytroblastosis<br>-Increase in palpable splenomegaly of >5 cm from baseline or the development of a newly palpable splenomegaly<br>-Lactate dehydrogenase level above the reference range

Table S4: Diagnostic criteria for essential thrombocythaemia (**A**) and for post–essential thrombocythaemia myelofibrosis (post-ET MF) (**B**) according to WHO guidelines [7].

A

Major criteria:

- Platelet count ≥ 450 × 10^9/L
- Bone marrow biopsy showing proliferation mainly of the megakaryocytic lineage, with increased numbers of enlarged, mature megakaryocytes with hyperlobulated nuclei; no significant increase or left shift in neutrophil granulopoiesis or erythropoiesis; very rarelya minor (grade 1) increase in reticulin fibres
- WHO criteria for *BCR*::*ABL1*-positive chronic myeloid leukaemia (CML), polycythaemia vera, primary myelofibrosis, and other myeloidneoplasms are not met
- *JAK2*, *CALR*, or *MPL* mutation

Minor criteria:

- Presence of a clonal marker

OR

- Exclusion of reactive thrombocytosis

The diagnosis of essential thrombocythaemia requires either all the major criteria or the first three major criteria plus a minor criterion to be met.

B

Required criteria:

- Documentation of a previous diagnosis of WHO-defined essential thrombocythaemia
- Bone marrow fibrosis of grade 2–3 on a scale of 0–3

Additional criteria:

- Anaemia (below the reference range, given age, sex, and altitude considerations) and a > 2 g/dL decrease from baseline haemoglobinconcentration
- Leukoerythroblastosis
- Increasing splenomegaly, defined as either an increase in palpable splenomegaly of > 50 mm from baseline (distance from the left costalmargin, or on imaging) or the development of newly palpable splenomegaly
- Elevated LDH level (above the reference range)
- Development of any two (or all three) of the following constitutional symptoms: > 10% weight loss in 6 months, night sweats,unexplained fever (> 37.5°C)

The diagnosis of post-ET MF requires either all the required criteria or at least two additional criteria.

Table S5: Diagnostic criteria for primary myelofibrosis, prefibrotic (**A**) and for primary myelofibrosis, fibrotic stage (**B**) according to WHO guidelines [7].

A

Major criteria:

- Megakaryocytic proliferation and atypia, without reticulin fibrosis grade >1, accompanied by increased age-adjusted bone marrowcellularity, granulocytic proliferation, and (often) decreased erythropoiesis
- Not meeting diagnostic criteria for chronic myeloid leukaemia, polycythaemia vera, essential thrombocythaemia, myelodysplasticneoplasms, or other defined myeloid neoplasms
- *JAK2*, *CALR*, or *MPL* mutation or presence of another clonal marker or absence of reactive bone marrow fibrosis

Minor criteria:

- Anaemia not attributed to a comorbid condition
- Leukocytosis $\geq 11 \times 10^9$/L
- Splenomegaly detected clinically and/or by imaging
- LDH level above the upper limit of the institutional reference range

The diagnosis of prefibrotic primary myelofibrosis requires all three major criteria and at least one minor criterion to be confirmed in two consecutive determinations.

B

Major criteria:

- Megakaryocytic proliferation and atypia, accompanied by reticulin and/or collagen fibrosis grade 2 or 3
- Not meeting diagnostic criteria for chronic myeloid leukaemia, polycythaemia vera, essential thrombocythaemia, myelodysplasticneoplasms, or other defined myeloid neoplasms
- *JAK2*, *CALR*, or *MPL* mutation or presence of another clonal marker or absence of reactive bone marrow fibrosis

Minor criteria:

- Anaemia not attributed to a comorbid condition
- Leukocytosis $\geq 11 \times 10^9$/L
- Splenomegaly detected clinically and/or by imaging
- LDH level above the upper limit of the institutional reference range
- Leukoerythroblastosis

The diagnosis of overt primary myelofibrosis requires all three major criteria and at least one minor criterion to be met in two consecutive determinations.

Table S6: Diagnostic criteria for for polycythaemia vera (**A**) and for for post–polycythaemia vera myelofibrosis (post-PV MF) (**B**) according to WHO guidelines [7].

A

Major criteria:

1. Elevated haemoglobin concentration (> 16.5 g/dL in men, > 16.0 g/dL in women) or elevated haematocrit (> 49% in men, > 48% in women)
2. Bone marrow biopsy showing age-adjusted hypercellularity with trilineage growth (panmyelosis), including prominent erythroid, granulocytic, and megakaryocytic proliferation with pleomorphic, mature megakaryocytes (differences in size)
3. Presence of *JAK2* p.V617F or *JAK2* exon 12 mutation

Minor criterion:

- Subnormal serum erythropoietin level

The diagnosis of polycythaemia vera requires either all three major criteria or the first two major criteria plus the minor criterion

B

Required criteria:

- Documentation of a previous diagnosis of WHO-defined polycythaemia vera
- Bone marrow fibrosis of grade 2–3 on a scale of 0–3

Additional criteria (two are required):

- Anaemia (below the reference range, given age, sex, and altitude considerations) or sustained loss of requirement of either phlebotomy (inthe absence of cytoreductive therapy) or cytoreductive treatment for erythrocytosis
- Leukoerythroblastosis
- Increasing splenomegaly, defined as either an increase in palpable splenomegaly of >50 mm from baseline (distance from the left costalmargin) or the development of a newly palpable splenomegaly
- Development of any two (or all three) of the following constitutional symptoms: >10% weight loss in 6 months, night sweats,unexplained fever (> 37.5°C)
